# Effects of phosphorous and antimony doping on thin Ge layers grown on Si

**DOI:** 10.1038/s41598-024-57937-8

**Published:** 2024-04-04

**Authors:** Xueying Yu, Hui Jia, Junjie Yang, Mateus G. Masteghin, Harvey Beere, Makhayeni Mtunzi, Huiwen Deng, Suguo Huo, Chong Chen, Siming Chen, Mingchu Tang, Stephen J. Sweeney, David Ritchie, Alwyn Seeds, Huiyun Liu

**Affiliations:** 1 Department of Electronic and Electrical Engineering, University College London, Torrington Place, London, WC1E 7JE UK; 2 Advanced Technology Institute, University of Surrey, Guildford, Surrey GU2 7XH UK; 3 Cavendish Laboratory, University of Cambridge, Cambridge, CB3 0HE UK; 4 London Centre for Nanotechnology, 17-19 Gordon Street, London, WC1H 0AH UK; 5 James Watt School of Engineering, University of Glasgow, Glasgow, G12 8LT UK

**Keywords:** Nanoscale materials, Silicon photonics, Semiconductors

## Abstract

Suppression of threading dislocations (TDs) in thin germanium (Ge) layers grown on silicon (Si) substrates has been critical for realizing high-performance Si-based optoelectronic and electronic devices. An advanced growth strategy is desired to minimize the TD density within a thin Ge buffer layer in Ge-on-Si systems. In this work, we investigate the impact of P dopants in 500-nm thin Ge layers, with doping concentrations from 1 to 50 × 10^18^ cm^−3^. The introduction of P dopants has efficiently promoted TD reduction, whose potential mechanism has been explored by comparing it to the well-established Sb-doped Ge-on-Si system. P and Sb dopants reveal different defect-suppression mechanisms in Ge-on-Si samples, inspiring a novel co-doping technique by exploiting the advantages of both dopants. The surface TDD of the Ge buffer has been further reduced by the co-doping technique to the order of 10^7^ cm^−2^ with a thin Ge layer (of only 500 nm), which could provide a high-quality platform for high-performance Si-based semiconductor devices.

## Introduction

The development of Si-based optical semiconductor devices, benefiting from their compatibility with Complementary Metal Oxide Semiconductors (CMOS) technology, is highly motivated to validate Si-based optoelectronic integrated circuits (OEIC) and photonic integrated circuits (PIC)^1–8^. Nevertheless, Si suffers from its indirect bandgap, making it unreliable as an on-chip light emitter. Therefore, the direct growth of materials with superior optical properties on Si platforms has been explored in the past decades. As another group IV material, Ge has a pseudo-direct bandgap of 0.664 eV and an achievable direct bandgap of 0.8 eV, corresponding to the 1.55 μm communication band. However, it requires bandgap, strain and carrier density engineering to achieve lasing behaviour for Ge, making Ge lasers inefficient, uncompetitive and impractical compared to their III–V counterparts^9^. Heteroepitaxial growth of III-V compounds on Si has become another promising candidate to realize light-emitting sources on Si for further OEIC or PIC development^10^. To resolve the lattice mismatch between III–Vs and Si, a conventional thick GaAs buffer layer followed by dislocation filter layers (DFLs) has been employed to limit the threading dislocation density (TDD) in the active region^11–13^. However, thick III-V structures grown on Si would suffer from thermal cracks during the cooling process and bring difficulties to the integration of III-V lasers with the rest of the optical components, due to the difference in the thermal expansion coefficients of GaAs and Si. Thanks to the lattice similarity between Ge and GaAs, Ge has become an alternative buffer material for the monolithic integration of GaAs and Si. A thin Ge buffer layer could be utilised to replace the thick GaAs buffer layer for more efficient TDD reduction, thereby keeping the total thickness of the laser structure below the cracking threshold^14^. Direct epitaxy of Ge on Si would act as a “virtual substrate” (VS) for the growth of III-Vs and GaAs/InAs QDs active regions, which provides a potential solution for the practical integration of GaAs/InAs QD lasers and Si substrates^15–17^. High-quality Ge grown on Si would also benefit the demonstration of Ge photonic components, especially lasers and photodetectors^18–22^. However, the main challenge remains to be the large lattice mismatch (4.2%) between Ge and Si, which inevitably induces large and accumulative compressive strain during the epitaxial growth. The build-up strain relaxes forming three-dimensional (3D) islands or generating misfit dislocations at the interface when the deposition layer exceeds the critical thickness. The misfits are associated with threading dislocations (TDs) penetrating through the device structure and degrading its performance^23,24^.

Various approaches have been studied to improve the crystalline quality of Ge epitaxial layers. Thick (~ 10 s of microns) compositional graded SiGe buffer layers were used to reduce the TDD to as low as 10^6^ cm^−2^ by relaxing the Ge epilayer in a controlled manner^25^. However, structures with such thickness are not applicable in Si-based photonics integration, as it is both time- and cost-consuming and increases the possibility of thermal cracking. On the other hand, the ‘two-step’ growth is mostly used both by molecular beam epitaxy (MBE) and chemical vapour deposition (CVD) methods, where a layer grown at low temperature (LT) is first introduced to facilitate layer-by-layer growth, followed by a layer grown at high temperature (HT) to improve the crystal quality^16^. In addition to ‘two-step’ growth, introducing dopants to the LT layer has reported positive effects on suppressing TDs^26,27^. Due to the local strain induced by the doping atoms in the LT nucleation layer, existing TDs are promoted and interact with each other to trigger self-annihilation^28^. Yang et al. proposed a two-step growth method with doping techniques followed by a cyclic annealing process, bringing the TDD to 2.6 × 10^8^ cm^−2^ in a 500-nm Ge epilayer^14^. Other approaches, including selective epitaxial growth (SEG), have been attempted with the aim of a ‘defect-free’ Ge-on-Si system^29–31^. However, wafer-sized high-quality Ge film directly growing on Si remains challenging.

Employing n-type dopants such as Antimony (Sb) and Arsenic (As) during Ge growth is a reported successful strategy to reduce the threading dislocation density (TDD) and smoothen the Ge surface. Phosphorous (P) doping has also demonstrated a reduction in TDD compared with undoped and B-doped Ge on Si growth, which was attributed to the faster dislocation motion speed brought by the presence of shallow donor levels at the position of defects and the gettering effect of impurity atoms that makes the dislocations immobilized. Enhanced Ge/Si interdiffusion has also been reported for As and P doped samples and explained to be a result of Fermi level effect^32^. However, in the literature, more focus is on the achievement of uniform and high doping concentrations, less attention has been paid to the effect of dopants on TDD reduction. Here, we investigate the impact of different concentrations of P dopants on TDD reduction and surface morphology improvement in Ge layers monolithically grown on Si (001) substrates. The optimum doping concentration of P is determined, along with a discussion on the mechanism of the effect of the P dopants. For comparison, Sb-doped samples with varied doping densities are also grown and examined. P and Sb dopants have revealed different impacts on the growth of Ge on Si. Different from the well-known surfactant effect of Sb, P dopants affect the Ge growth in terms of enhancing Si-Ge interdiffusion, which is induced by the fast transport of P towards the Ge/Si interface^33,34^. Based on our understanding of Sb and P doping mechanisms, a novel co-doping technique is proposed and demonstrated to further reduce defects in the thin Ge buffer layer grown on Si. Several combinations of P and Sb concentrations have been attempted to optimize the Ge structural properties, and the lowest TDD for a 500-nm Ge layer has ultimately reached the order of 10^7^ cm^−2^, along with a low roughness root mean square (RMS) of 0.7 (with a standard deviation of 0.199) nm.

## Results

**Figure 1:**
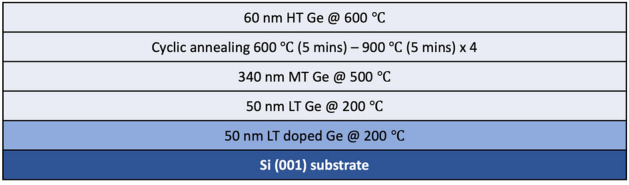
Schematic structure of Ge buffer layers grown on Si substrates.

**Figure 2:**
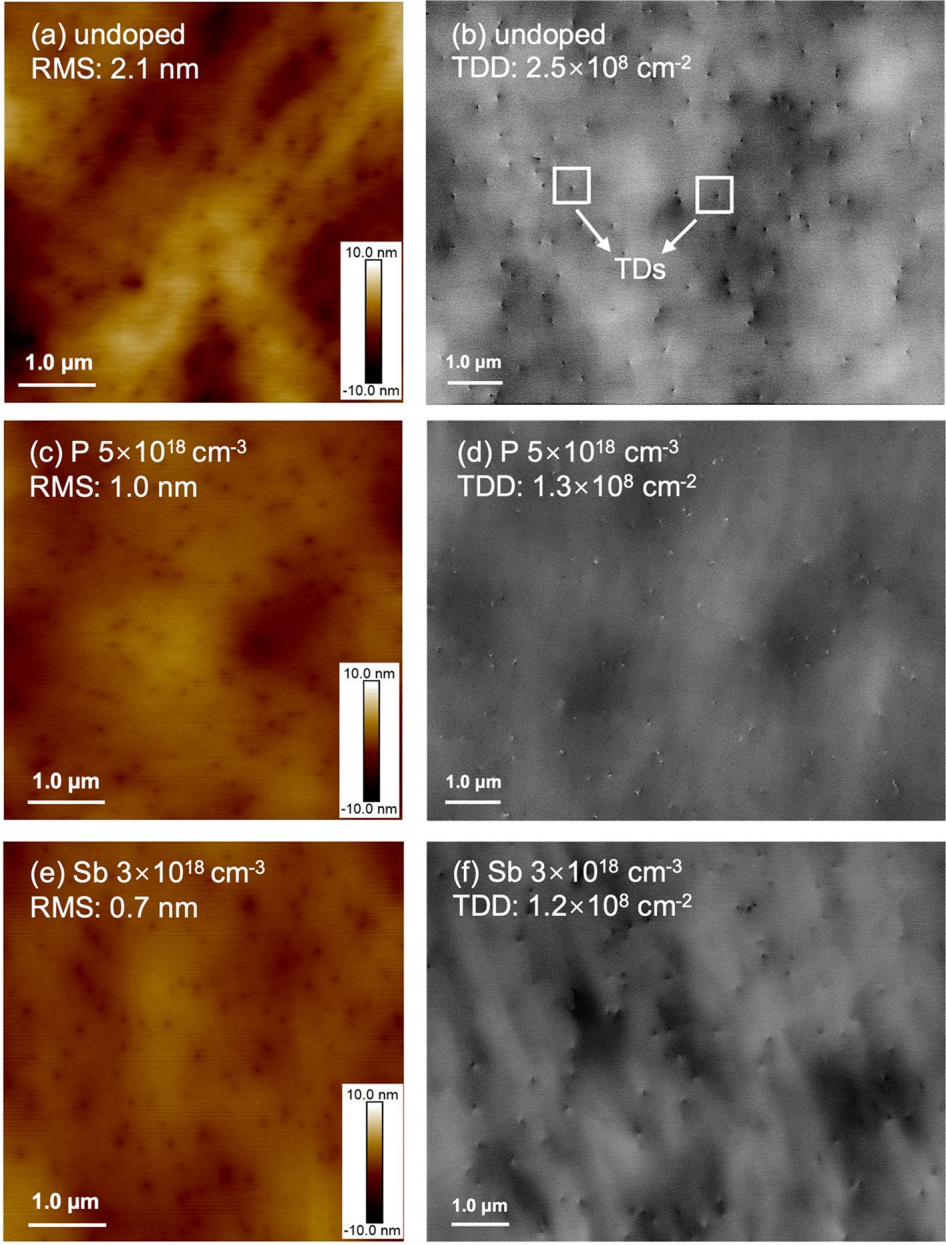
(**a**) 5 μm × 5 μm AFM image and (**b**) ECCI scan of the Ge sample with intrinsic LT Ge layer, (**c**) 5 μm × 5 μm AFM image and (**d**) ECCI scan of the Ge sample with 5 × 10^18^ cm^−3^ P-doped LT Ge layer, (**e**) 5 μm × 5 μm AFM image and (**f**) ECCI scan of the Ge sample with 3 × 10^18^ cm^−3^ Sb-doped LT Ge layer. The white squares indicate the TDs detected by the ECCI scan.

**Figure 3:**
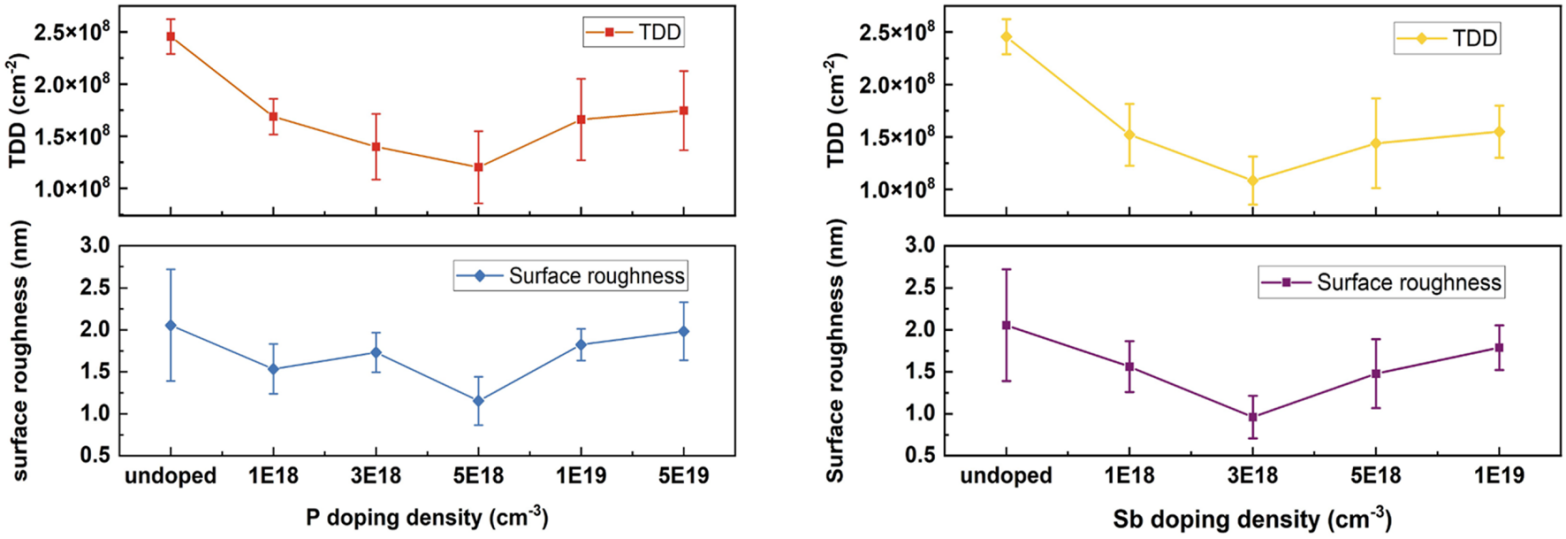
Summarized plots of TDD and RMS roughness of samples with (**a**) P doping density of 0, 1, 3, 5, 10, and 50 × 10^18^ cm^−3^, indicating the lowest TDD occurs at P concentration of 5 × 10^18^ cm^−3^ and (**b**) Sb doping density of 0, 1, 3, 5 and 7 × 10^18^ cm^−3^, indicating the lowest TDD occurs at Sb concentration of 3 × 10^18^ cm^−3^.

Benefiting from the high solubility of P atoms in Ge, a heavier doping density of P can be achieved compared to Sb and As^35^. In this work, a wide range of P doping densities of 1 to 50 × 10^18^ cm^−3^ have been attempted in the growth structure indicated in Fig. 1. Note that only the initial LT Ge layer was in-situ doped by different dopants, and the rest of the Ge structure was intrinsic. A controlled sample with an undoped Ge LT layer has been prepared as a reference for the Ge epi-layer quality analysis. The surface morphologies of the undoped and the 5 × 10^18^ cm^−3^ P-doped samples are investigated by AFM and ECCI scans, as shown in Fig. 2a–d. The AFM measurements indicate a decrease in surface roughness from 2.1 to 1.0 nm in the Ge sample with P doping. In the meantime, the ECCI images in Fig. 2c, d show the surface TDs of the undoped and the P-doped Ge samples, where the TDs are indicated as pits highlighted by the white squares. The measured TDD is nearly halved with P doping, decreasing from 2.5 × 10^8^ to 1.3 × 10^8^ cm^−2^ according to Fig. 2c, d. A summarised plot is shown in Fig. 3a to explain the impact of different P doping densities on the Ge surface morphology. The data was evaluated by averaging multiple measurements on different positions of different samples (centre, intermediate, edge area), and the error bar was calculated by standard deviation in both (a) and (b). The varying trends of the TDD and RMS roughness are given with respect to P concentration, based on which the optimum doping density of P is determined as 5 × 10^18^ cm^−3^. As the P concentration exceeds 5 × 10^18^ cm^−3^, the Ge sample exhibits a higher TDD.

To compare P with other n-type dopants, a group of Sb-doped samples with doping densities from 1 to 7 × 10^18^ cm^−3^ have been grown and analyzed. The surface roughness of the undoped and the 3 × 10^18^ cm^−3^ Sb-doped samples are compared in Fig. 2a, e, indicating a reduction from 2.1 to 0.7 nm. The surface TDD of the two samples are exhibited in Fig. 2b, f, which reveal that the TDD of the Ge sample with Sb doping is lowered by more than half, reaching a value of 1.2 × 10^8^ cm^−2^. The impact of different Sb doping densities on both TDD and RMS surface roughness is summarized in Fig. 3b. Both surface roughness and TDD of the Ge sample decline with increasing Sb concentrations and reach the lowest at Sb density of 3 × 10^18^ cm^−3^, which is hence determined as the optimized doping density of Sb. Sb-mediated Ge layer growth on Si has been well studied previously, demonstrating improvements in Ge quality, which are attributed to the well-known surfactant effect of Sb^36,37^. Sb atoms are reported to be efficient when passivating the atomic steps on the Si surface. The Ge adatoms will, therefore, not be able to distinguish between step edges and flat regions during deposition, which reduces the island nucleation and results in high structural quality^38^. In addition, the atomic radius of Sb is 16% larger than that of Ge, which introduces a local strain to the Ge crystal. TDs can easily get deflected by point defects and local strain followed by an interaction with other dislocations, which is known as the TD annihilation process^39^. However, according to Fig. 3b, an increase in roughness and TDD occurs during further increments of Sb concentration, illustrating that heavy Sb doping introduces degraded surface smoothness and higher TDD. As the concentration of Sb increases further, the induced strain builds up and can introduce additional defects in the structure. Heavily doped Sb atoms are also more likely to segregate from the Ge layer and form clusters during growth due to limited solid solubility, leading to insufficient doping and large defective clusters in the structure^40^.

**Figure 4:**
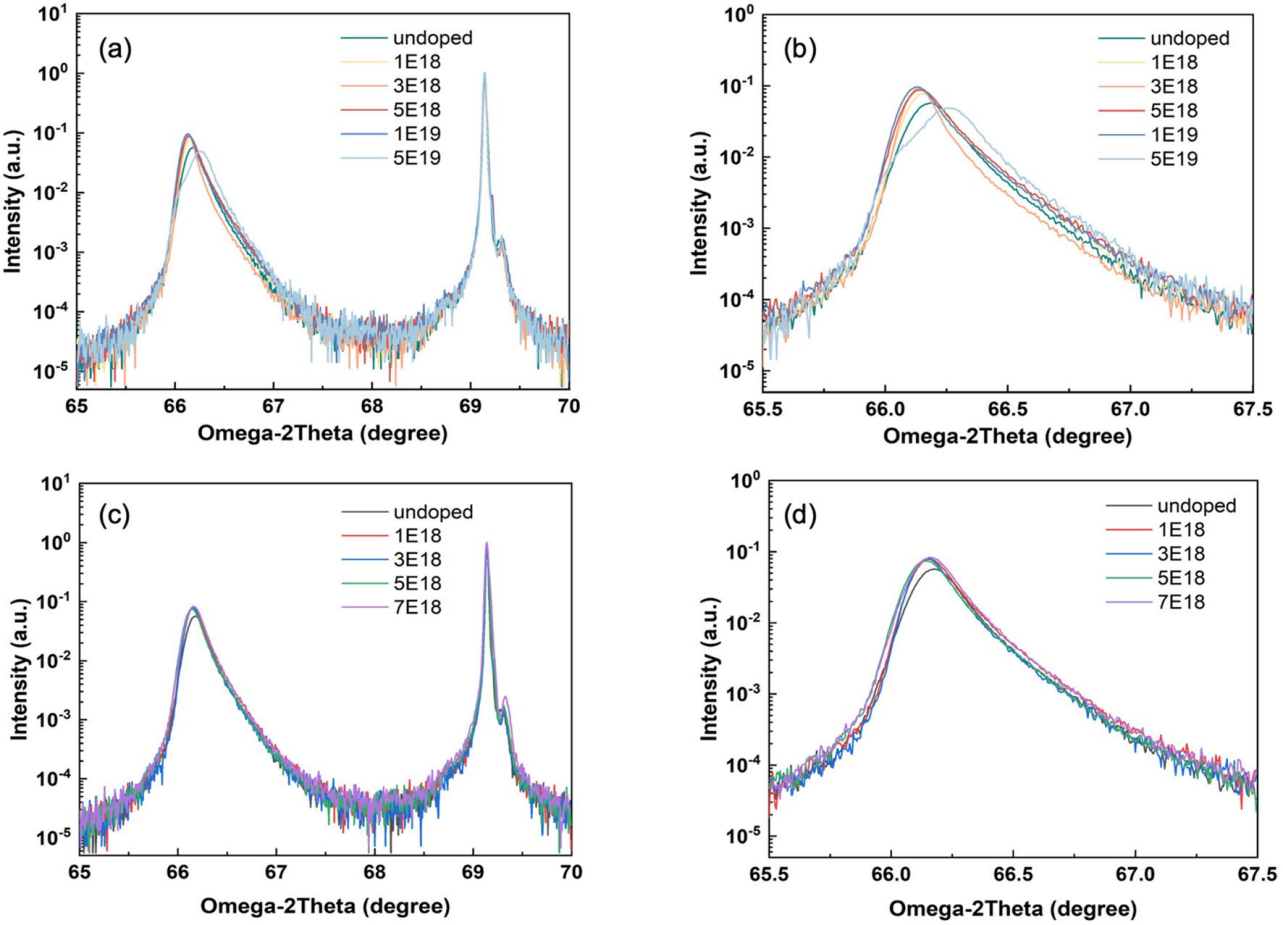
(**a**) HR-XRD 2theta-omega measurements on the (004) plane of samples with P doping density of 0, 1, 3, 5, 10, and 50 × 10^18^ cm^−3^, (**b**) enlarged Ge peaks of the P-doping HR-XRD, indicating the Ge peak shifts to smaller angles with increasing doping density, (**c**) HR-XRD 2theta-omega measurements of samples on the (004) plane with Sb doping density of 0, 1, 3, 5 and 7 × 10^18^ cm^−3^ and (**d**) enlarged Ge peaks of the Sb-doping HR-XRD, indicating consistent Ge peaks.

Since comparable TD suppression has been depicted by P doping and Sb doping, it is important to investigate the mechanism of the P doping effect and contrast it with the Sb doping technique. HR-XRD measurements for both P and Sb-doped Ge samples are performed to evaluate the relaxation of the Ge layers. Figure 4a–d are the two theta-omega scans on the (004) plane of the Ge on Si samples. Figure 4a presents the HR-XRD results for the P-doped samples, with the enlargement of the Ge peaks depicted in (b). A continuous shift of the Ge peaks away from the normalized Si peak can be observed with the increasing P concentration, indicating that P doping has enhanced the out-of-plane strain relaxation in the growth direction of the Ge layers. Table 1 is calculated based on the Ge peak position in Fig. 4, illustrating the expansion of lattice constant of the Ge epilayer under the impact of the increasing P doping density. The small peak on the right side of the main Si-substrate peak in Fig. 4a, c can be attributed to the resolution of the single reflection from 004 planes into two peaks due to K_α1_ and K_α2_ rays^41,42^.

**Table 1:** A summary of the lattice constants calculated from the peak positions from the HRXRD results and peak widths of samples.

Material	Lattice constant calculated from peak (Å)	Peak width (°)
Si	5.430	0.029
Ge:undoped	5.643	0.188
Ge:P 1E18	5.645	0.170
Ge:P 3E18	5.646	0.132
Ge:P 5E18	5.647	0.173
Ge:P 1E19	5.647	0.160
Ge:P 5E19	5.653 (left)/5.638 (right)	0.225 (peak on the right)

From Table 1 it can be concluded that as the P doping density increases, the Ge peak shifts less and finally splits at the doping density of 5 × 10^19^ cm^−3^, indicated as the light blue line in Fig. 4b. The peak appearing on the left of the splitting Ge peak is close to bulk Ge lattice constant (5.657 Å), while the one on the right may be attributed to the GeSi alloy formed by possible Si-Ge interdiffusion^43^. The HR-XRD measurement suggests that excessive P dopants do not contribute to further relief of the epilayer strain. Considering the AFM and ECCI results introduced previously, excessively heavy doping may not favour the improvement of the Ge layer. Figure 4c, d present the HR-XRD measurements of the Sb-doped samples. The Ge peak is slightly offset between doped and undoped samples due to strain induced by Sb dopants. However, increasing Sb doping densities does not further shift the Ge peak, illustrating that the strain release of the Ge layer is not significantly affected by the increased concentration of Sb dopants, up to 7 × 10^18^ cm^−3^. A higher concentration of Sb has been attempted, but severe segregation of Sb was observed during the growth, which made the sample unsuitable to characterise. In addition, the Sb-doped sample started to degrade as the Sb doping density went beyond 5 × 10^18^ cm^−3^ and further. Therefore, the highest concentration of Sb in this work was chosen to be slightly lower than that of P. The HR-XRD results imply that the TDD reduction induced by P doping is likely to differ from that by Sb doping, which will be further explored by SIMS and TEM measurements.

**Figure 5:**
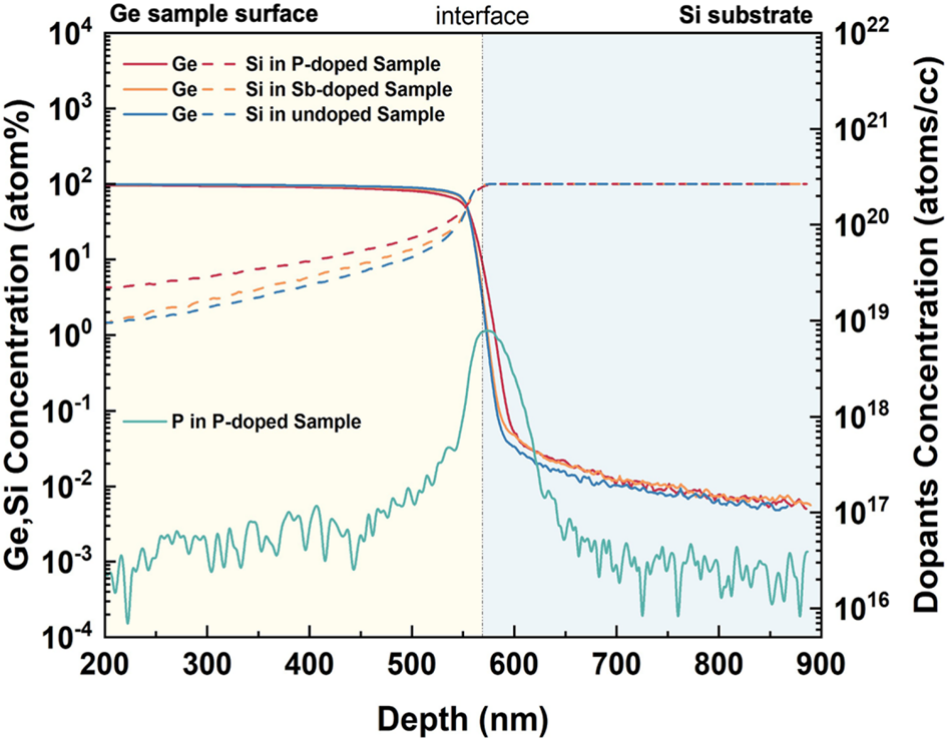
SIMS results describe the varied concentration of compositions in the 5 × 10^19^ cm^−3^ P-doped, the 3 × 10^18^ cm^−3^ Sb-doped, and the undoped samples, respectively.

To understand the doping profile in the samples, SIMS measurements of the P-doped (5 × 10^19^ cm^−3^), Sb-doped (3 × 10^18^ cm^−3^), and the undoped reference sample have been carried out and displayed in Fig. 5 to provide composition information of the Ge-on-Si structures. Note the dopant profile was measured together with Ge and Si. A standard sample with the same composition as the analytical sample is used for removing the matrix effect and ensuring accurate quantification. For the 5 × 10^19^ cm^−3^ P-doped sample, the peak of the P concentration curve (green) is observed near the Ge/Si interface, with some P atoms detected in the Si substrate. This reveals that the P dopants tend to migrate towards the Si substrate and gather at the Ge/Si interface. The Ge and Si concentration profiles in the P-doped sample are indicated by the red straight line and red dotted line, respectively, which illustrate severe interdiffusion of Ge and Si compared to the other two samples. Si atoms diffuse throughout the Ge structure with the Si concentration gradually decreasing, forming a graded GeSi region that may favour the strain relaxation of the Ge epilayer.

The transport of P dopants in Ge-on-Si systems has been previously investigated^44,45^. These studies suggest that P atoms experience rapid diffusion towards the high-defect density regions, i.e., the Ge/Si interface^46^. The diffusivity of P increases dramatically at the high concentration (high 10^18^ cm^−2^ and above), which can be attributed to the phosphorous-vacancy pairs introduced in Ge during P implementation^47–50^. The concentration-enhanced P transport consequently promotes the interdiffusion of Ge and Si, which can be explained by considering the Fermi effect^33,51^. High P concentration accelerates the Ge-Si interdiffusivity and results in a thin layer of GeSi alloy, acting as a graded GeSi layer between the Ge epi-layer and Si substrate^34^. The difference in TD velocities in the Si-diffused region and the ‘undoped’ region gives rise to the bending of TDs and eventually TDs can form pure edge dislocations^52–55^. For the remaining 60° TDs, with opposite Burgers vectors, they can react and disappear from the surface during the high-temperature cyclic annealing process. In our experiment, the Si-diffusion into Ge is enhanced by n-type dopants, which might have provided a reasonable clue for the observed reduction in TDD.

**Figure 6:**
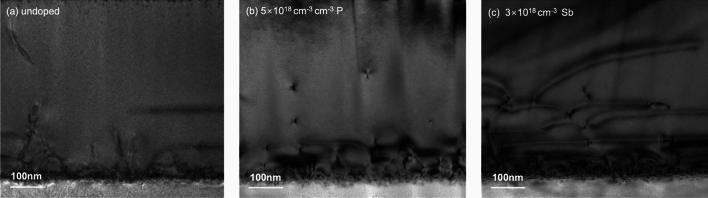
Cross-sectional TEM images presenting the TDs at the Ge/Si interfaces of the (**a**) undoped sample, (**b**) P-doped sample and (**c**) Sb-doped sample.

We then focus on the TD morphology at the Ge/Si interface by including the cross-sectional TEM measurement, which monitors the initial TD generation and propagation under the influence of P and Sb doping. As shown in Fig. 6, a large number of defects can be observed at all sample interfaces with associated TDs, most of which are confined within approximately the first 200 nm growth. The most impressive observation is the distinctive interface and TD morphology provided by different dopant incorporations in the samples. The undoped sample is presented in Fig. 6a, where high density of defects can be found at the interface. With the absence of dopants, the formed TDs propagate freely and mainly vertically towards the surface. Compared to Fig. 6a, the P-doped sample represented by Fig. 6b exhibits a rough and non-flat Ge/Si interface. In contrast, Fig. 6c demonstrates a defined and flat Ge/Si interface in the Sb-doped sample, thanks to the superior surfactant effect of Sb. On the other hand, a smaller amount of TD generation and propagation can be observed in the P-doped sample. P atoms may contribute to the relaxation of Ge epilayers in a way that fewer defects are produced, resulting in fewer TDs observed in the initial LT-grown region. This low-density dislocation generation could be attributed to the graded buffer layer produced by the Ge–Si intermixing, which allows a slow and controlled strain relief in the lattice-mismatched epilayer without further dislocation formation^56^. In this case, the strain release would favour the gliding of the existing TDs to the edge of the sample^57,58^. The initially-grown region of the Sb-doped sample, in contrast, exhibits more TDs that form a large network, from which Sb-enhanced TD bending can be clearly observed.

**Figure 7:**
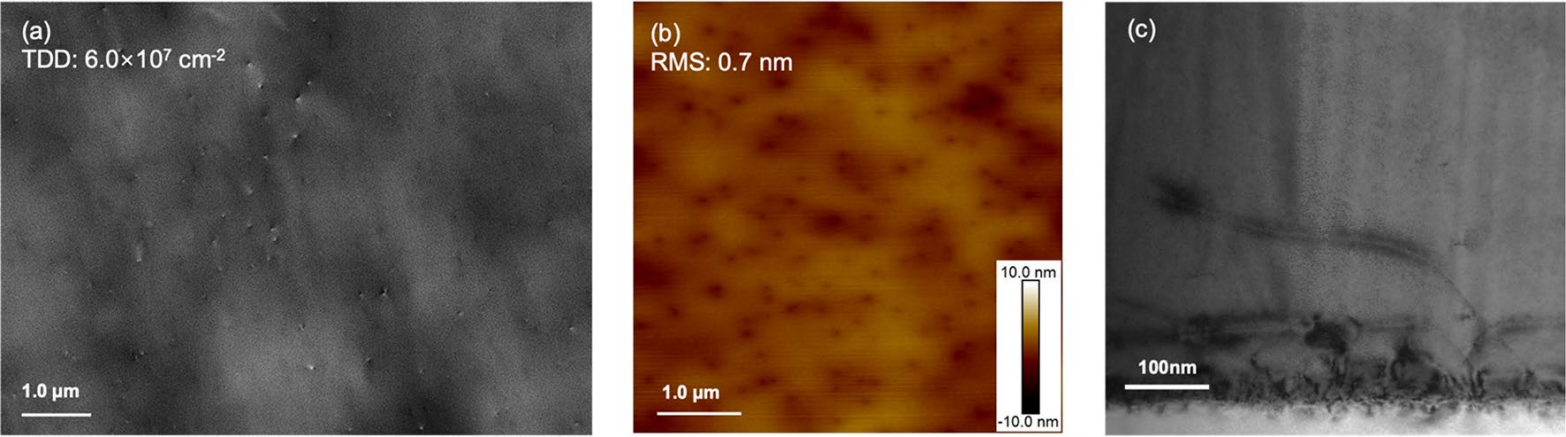
(**a**) ECCI scan describing the surface TDD, (**b**) 5 μm × 5 μm AFM image showing the surface morphology of the sample with co-doping of 1 × 10^18^ cm^−3^ P and 3 × 10^18^ cm^−3^ Sb and (**c**) cross-sectional TEM image presenting the TDs at the Ge/Si interface of the co-doped sample.

Sb and P doping have both demonstrated positive impacts on improving the Ge layer quality but undergo slightly different working mechanisms. TDD suppression by Sb doping is mainly through the TD nucleation stage and the local-strain-induced TD bending, while P doping promotes significant Si diffusion into Ge and enables a Si gradient at the Ge/Si interface that can bend the TDs to the wafer edge. Therefore, it is expected that the incorporation of these two n-type dopants would exploit the advantages from both Sb and P doping effects. It has been reported that the co-doping of Sb and P can compensate the dopant-related stress and increase the substitutional solubility of both dopants and free electron concentration^59^. This change will react on the number of double negatively charged dopant-vacancy pairs and thus the dopants diffusion and TD dynamics. A novel co-doping technique has therefore been proposed by employing both P and Sb dopants in the LT Ge layer simultaneously. The growth structure is identical to the one depicted in Fig. 1. Different combinations of P and Sb doping concentrations have been attempted. The best result has been obtained with 3 × 10^18^ cm^−3^ of Sb and 1 × 10^18^ cm^−3^ of P. By introducing this combination of P and Sb dopants, the TDD of a 500 nm Ge buffer layer has been significantly reduced by more than a half compared to the undoped sample, with a low surface roughness of 0.7 (± 0.04) nm, as displayed in Fig. 7a, b. A reference pair of co-doped and undoped Ge samples with thickness of 1 μm was also prepared, demonstrating a reduction in TDD by approximately an order of magnitude. More than ten times of repetition proves that this result is repeatable. Figure 7c exhibits the cross-sectional TEM of the co-doped sample, presenting a reduced TD network in the initial deposition region compared to the singly-Sb-doped sample, along with a rather fluctuating Ge/Si interface. We therefore perform a SIMS measurement on the co-doped sample, and the result suggests that the intermixing of Ge and Si also exists, which indicates that the co-doped Ge layer also benefits from the P-induced GeSi grading. Both TEM and SIMS measurements illustrate that the initially generated dislocations in the co-doped sample could have been impeded by the Ge-Si intermixing promoted by the P dopants. The use of capping layers is also reported to have an impact on the near-surface distribution of intrinsic point defects and the out-diffusion of the dopant atoms. The formation energy of self-interstitial and vacancies is considerably lowered close to the wafer surface^59^. In our experiment, the second LT Ge layer can be regarded as a capping layer that should function as a diffusion barrier for the dopants underneath. In addition, Sb dopants would have an extra effect of enhancing the TD annihilation and smoothening the sample surface, permitting a Ge sample with significantly improved quality.

## Conclusions

**Figure 8:**
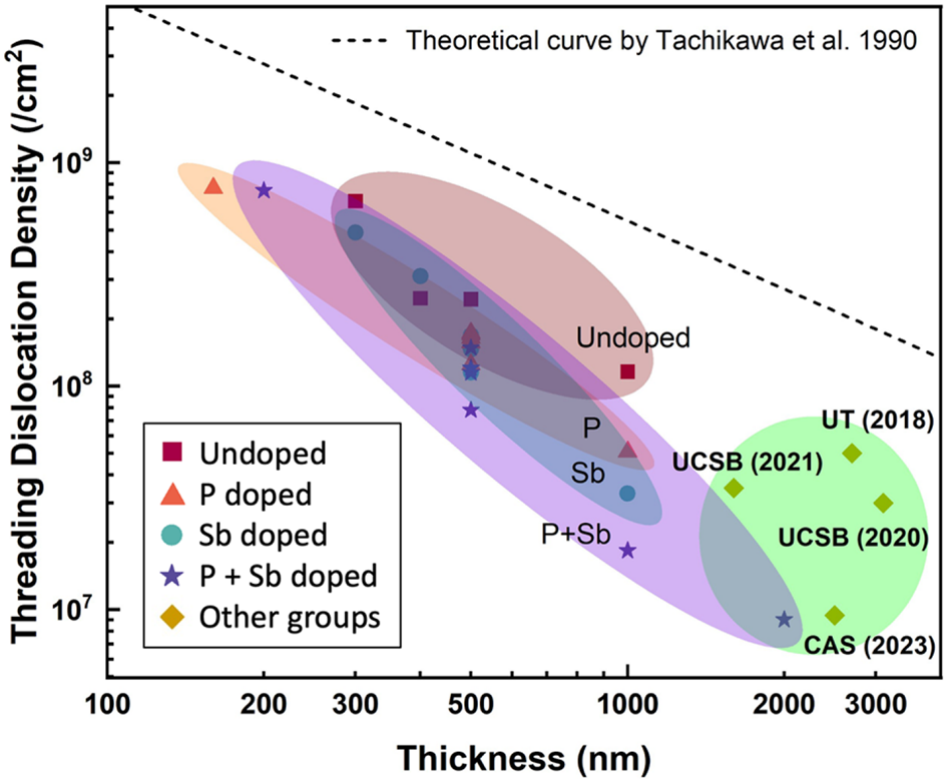
TDD vs. epitaxial layer thickness. The data from this work are compared to recent results from GaAs/Si reported by other groups and the theoretical estimation curve of TDD with increased epilayer thickness.

The optimised thin Ge buffer layer can serve as a high-quality platform to replace part of the conventional thick GaAs buffer layer employed in the InAs/GaAs QD laser structures on Si. Figure 8 compares the ECCI-measured TDD of the Ge films in this work and the most recent results of GaAs layers reported by other groups. The data are plotted as a function of layer thickness, including a theoretical line estimating the TDD reduction with increased epilayer thickness^39,60–63^. It demonstrates an improvement in TDD reduction by our doping techniques. P doping technique has been revealed to be an effective method to improve the quality of the Ge buffer layer grown on Si by impeding the dislocation formation in the initial stage of growth. This phenomenon was attributed to the Si-Ge interdiffusion induced by the fast transport of P towards the Ge/Si interface. From a series of Ge samples with a large range of P doping densities, the optimized doping concentration of P has been determined, which halves the TDD and improves the surface flatness of the Ge epilayer. A group of Sb-doped Ge samples with identical growth structures to the P-doped ones has also been prepared for comparison between P and Sb dopants. Sb-doped samples suggested an improvement in Ge surface smoothness, as well as a significant suppression in TD propagation, thanks to the surfactant effect of Sb. P and Sb doping demonstrated comparable improvement on Ge buffer layers under different working mechanisms. Accordingly, a novel co-doping approach has been attempted and investigated. Benefitting from the Si–Ge intermixing enhanced by P diffusion and the TD annihilation promoted by Sb, the quality of the Ge layer has been improved. A dramatic reduction of TDD to the level of 10^7^ cm^−2^ and of surface roughness to 0.7 nm has been achieved in a 500-nm Ge buffer layer. This result demonstrates that the co-doping of Sb and P has the potential for growing high-quality Ge thin films on Si for the development of Si-based optoelectronic devices.

## Experimental section

The samples were grown in a Veeco Gen-930 solid-source MBE system, implemented with two Ge cells, a GaP cell, and an Sb cracker cell. All samples were grown on 3-inch Si wafers with 4˚ offcut towards <110>  ± 0.1°, which were first treated with a thermal deoxidization process to remove the native oxide. Ge layers were then grown on Si substrates using the two-step growth method followed by thermal cyclic annealing (TCA) treatment, as illustrated in Fig. 1. The initial LT seed layer was key in obtaining high-quality Ge epilayers, which was of interest in our doping experiments^35^. All doping configurations were carried out in this layer, with the rest of the Ge structure being intrinsic. The first 50-nm LT Ge layer was doped by P and/or Sb with different concentrations at a growth temperature of 200 °C. The growth temperature of the initial LT layer was kept low to prevent the 3D nucleation of Ge atoms and maintain the flatness of the surface. Another 50-nm LT intrinsic Ge layer was grown as the cap layer during the later temperature increment. Given the mismatching lattice of Ge and Si, the initial layers suffered the most from the strain and contained high-density defects. A mid-temperature (MT) layer was grown at 500 °C to constrain the propagation of the TDs formed in the LT layer and produce a stabilized crystalline structure^64^. A four-cycle TCA between 600 and 900 °C was executed in sequence to enhance the motion of TDs and promote their self-annihilations^65^. The structure was finished by a 60-nm HT cap layer grown at 600 °C to further smoothen the surface. The Ge samples were characterized by atomic force microscopy (AFM) and high-resolution X-ray diffraction (HR-XRD), providing information on the surface morphologies and structural strain, respectively. Electron channeling contrast imaging (ECCI) was involved to measure the TDD on the sample surface, and cross-sectional transmission electron microscopy (TEM) was carried out in a Thermo Scientific Talos F200i to describe the TD distribution and propagation within the Ge structures. A lamella was prepared using a Xenon plasma focused ion beam (pFIB), Tescan FERA3. Both bright-field and weak-beam dark-field modes [at the 3 g condition for the (220) plane] were performed at 200 keV. Secondary ion mass spectrometry (SIMS) measurements were performed for the composition analysis of the Ge-on-Si systems.

## Data Availability

The data that support the findings of this study are available from the corresponding author upon reasonable request.
